# Glucagon-like peptide-1 receptor agonists and the risk of cardiovascular events in diabetes patients surviving an acute myocardial infarction

**DOI:** 10.1093/ehjcvp/pvaa004

**Published:** 2020-01-30

**Authors:** Marco Trevisan, Edouard L Fu, Karolina Szummer, Anna Norhammar, Pia Lundman, Christoph Wanner, Arvid Sjölander, Tomas Jernberg, Juan Jesus Carrero

**Affiliations:** 1 Department of Medical Epidemiology and Biostatistics, Karolinska Institutet, Nobels väg 12A, 171 77 Stockholm, Sweden; 2 Department of Clinical Epidemiology, Leiden University Medical Center, Leiden, The Netherlands; 3 Department of Cardiology, Karolinska University Hospital, Solna, Sweden; 4 Department of Medicine, Karolinska Institutet, Huddinge, Sweden; 5 Department of Medicine, Karolinska Institutet, Solna, Sweden; 6 Capio Saint Görans hospital, Stockholm, Sweden; 7 Department of Clinical Sciences, Danderyd University Hospital, Karolinska Institutet, Stockholm, Sweden; 8 Department of Medicine, Würzburg University Clinic, Würzburg, Germany

**Keywords:** GLP-1 receptor agonist, SWEDEHEART, Myocardial infarction, Diabetes, Cardiovascular events

## Abstract

**Aims:**

Trial evidence indicates that glucagon-like peptide-1 receptor agonists (GLP-1 RAs) may reduce the risk of cardiovascular (CV) events in patients with diabetes and myocardial infarction (MI). We aimed to expand this observation to routine care settings.

**Methods and results:**

Prospective observational study including all patients with diabetes surviving an MI and registered in the nationwide SWEDEHEART registry during 2010–17. Multivariable Cox regression analyses were used to estimate the association between GLP-1 RAs use and the study outcome, which was a composite of stroke, heart failure, Re-infarction, or CV death. Covariates included demographics, comorbidities, presentation at admission, and use of secondary CV prevention therapies. In total, 17 868 patients with diabetes were discharged alive after a first event of MI. Their median age was 71 years, 36% were women and their median estimated glomerular filtration rate was 75 mL/min/1.73m^2^. Of those, 365 (2%) were using GLP-1 RAs. During median 3 years of follow-up, 7005 patients experienced the primary composite outcome. Compared with standard of diabetes care, use of GLP-1 RAs was associated with a lower event risk [adjusted hazard ratio (HR) 0.72; 95% confidence interval (CI): 0.56–0.92], mainly attributed to a lower rate of re-infarction and stroke. Results were similar after propensity score matching or when compared with users of sulfonylurea. There was no suggestion of heterogeneity across subgroups of age, sex, chronic kidney disease, and STEMI.

**Conclusion:**

GLP-1 RAs use, compared with standard of diabetes care, was associated with lower risk for major CV events in healthcare-managed survivors of an MI.

## Introduction

About 20–25% of patients admitted with a myocardial infarction (MI) in Europe have established diabetes mellitus.^1^ Patients with diabetes have long been known to be at high risk for morbidity and mortality after an MI,^2^^,^^3^ in part, because of more extensive coronary artery disease, additional cardiovascular (CV) risk factors, and higher burden of comorbidities.^4^^,^^5^ This increased CV burden is still present in diabetes patients with acute coronary syndrome (ACS), despite extensive use of modern evidence-based therapies.^5^ There is a need to improve secondary CV prevention strategies in these high-risk individuals.

Glucagon-like peptide-1 receptor agonists (GLP-1 RAs) are novel glucose-lowering treatments for type 2 diabetes with low risk for hypoglycaemia.^6^ Modest reductions in systolic blood pressure (BP), inflammation, and lipid concentrations as well as significant reduction in body weight have been observed in patients treated with GLP-1 RAs, and they have, therefore, been suggested as candidates for use in patients with diabetes at high risk of CV disease (CVD).^7^ Since the FDA requirements in 2008 of CV-safety data for novel diabetes agents, various trials have evaluated the efficacy and safety of GLP-1 RAs against placebo in diabetes patients with established CVD.^8–14^ While all of the trials showed CV safety (i.e. non-inferiority) over standard of care, some^8^^,^^9^^,^^12^^,^^13^ but not all^10^^,^^11^^,^^14^ observed efficacy in reducing their primary major adverse CV events (MACE) outcome (CV mortality, non-fatal MI, and non-fatal stroke). Differences across trials may be attributed not only to differences across GLP-1 RA agents, but also to differences in CV-risk profiles of included patients, including temporality of the event (prevalent vs. incident/acute CVD cases).

While trials assess drug safety and efficacy to gain regulatory approval, the rigours of trials can at times limit their generalizability to the larger population. Expanding trial evidence to observational studies from routine healthcare may offer complementary evidence to inform clinical decisions on the management of these patients. Against this background, we aimed to investigate, in a nationwide setting, the CV effectiveness associated to use of GLP-1 RAs at the time of an acute MI.

## Methods

### Data sources

We used data from SWEDEHEART (Swedish Web‐system for Enhancement and Development of Evidence‐Based Care in Heart Disease Evaluated According to Recommended Therapies), a nationwide registry of patients hospitalized for suspected ACS or undergoing coronary or valve intervention.^15^ All Swedish hospitals (*n* = 72) contribute data to SWEDEHEART, covering around 90% of patients with MI treated in hospitals in Sweden. The registry is monitored on a regular basis, with 95–96% agreement with regards to key variables between the registry and electronic health records. Rich patient information is collected prospectively, including patient demographics, past medical history, medical treatment before admission, electrocardiographic changes, clinical investigations, medical treatment in hospital, interventions, hospital outcome, diagnoses, and medication at discharge. Patients receive information about their participation in SWEDEHEART at admission and are allowed to opt out, but individual consent is not required. For this study, and via each citizen’s unique personal identification number, SWEDEHEART was enriched with data linkages with the Swedish Registry of Dispensed Drugs, which contains all pharmacy‐drug dispensations in the country since 2005, and the National Patient Registry, which includes all ICD-10 diagnoses issues in connection with an outpatient-specialist or inpatient consultation in Sweden since 1997. The study protocol was approved by the regional Institutional Review Boards and adhered to the Declaration of Helsinki.

### Study population

We included all adult (>18 years) survivors of an MI during 2010–17 with a diagnosis of diabetes mellitus and with a dispended glucose-lowering drug at the time of their MI, which was considered the index date of the study (Supplementary material online, *Figure S1*). We selected this period because GLP-1 RAs were introduced in Sweden in 2009. Exclusion criteria included previous history of MI, in-hospital death, and concomitant use of sodium-glucose transport protein-2 (SGLT-2) inhibitors.

### Study design, exposure, and covariates

The study exposure was GLP-1 RAs use vs. non-use (i.e. standard of diabetes care). Use of GLP-1 RAs was defined as a dispensation of this medication within 1 month after hospital discharge or 6 months prior to index event. Patients were considered on-treatment until death or end of follow-up (intention to treat analysis). Information on the use of other antidiabetic medications was also collected.

Study covariates included age, sex, smoking habits, and body mass index (BMI) as registered per SWEDEHEART protocol. Body mass index was considered a covariate because at the time of data collection GLP-1 RAs were subsidized in Sweden for type 2 diabetes patients with BMI >30 kg/m^2^. Estimated glomerular filtration rate (eGFR) was calculated with the 2009 CKD-EPI creatinine-based equation^16^ using plasma creatinine measured at hospital admission. Since information on race is not available in Sweden, we assumed all patients Caucasian.

Comorbidities included heart failure (HF), cancer, hypertension, stroke, peripheral vascular disease (PVD), and atrial fibrillation. Information on patient presentation and hospital course variables considered Killip class, ST-segment elevation MI (STEMI) and non-STEMI, percutaneous coronary intervention (PCI), and coronary artery bypass grafting (CABG). Information on secondary CV prevention medications dispensed at discharge [angiotensin-converting enzyme inhibitors (ACEi), angiotensin II receptor blockers (ARBs), β-blockers, statins, aspirin, and P2Y12-receptor blockers] was also collected.

### Study outcomes

The primary study outcome was the incidence of MACE, defined as the composite of non-fatal stroke, HF, or MI and death due to CV causes. Outcomes were ascertained by linkage with the Patient Register, which has complete coverage of hospitalization diagnoses and deaths in the country, with virtually no loss to follow-up. Hospitalizations occurring within the first 30 days post-discharge were attributed to the index event. Patients were followed until the occurrence of an event or censoring (i.e. non-CV death or end of follow-up), whichever occurred first.

### Statistical analysis

Baseline patient characteristics were described as median and interquartile range or counts with proportion. Unadjusted and adjusted cumulative incidence curves were estimated. The adjusted curves are obtained with regression standardization, which uses the predicted cumulative function of each patient and then averaged them over the observed distribution of the confounders in the population.^17^^,^^18^ Multivariable Cox proportional hazard models were used to estimate the association between GLP-1 RAs use and the risk of CV outcomes. We adjusted for clinical relevant variables known to influence both exposure and outcome, and then added other treatment known to influence outcome. Thus, the models were adjusted for age, sex, smoking, BMI, comorbidities [HF, stroke, PVD, cancer, hypertension, CKD (eGFR <60 mL/min/1.73 m^2^), PCI, CABG, atrial fibrillation, Killip class, and STEMI], and medication use (aspirin, statins, ACEi/ARBs, beta-blockers, and P2Y12-receptor blockers). Effect modification was investigated in the following strata defined a priori: age (< or ≥70 years), sex, eGFR category (eGFR < or ≥60 mL/min/1.73 m^2^), and STEMI. There were no missing values, except for BMI (missing in 6% of patients), smoking (missing in 7% of patients), and eGFR (missing in 3% of patients). Complete cases were considered for the analysis. The assumptions of proportionality and linearity (on the log hazard scale) in the Cox model were tested using Schoenfeld and martingale residuals, respectively. No relevant violation of this assumption was observed.

We performed several additional analyses to test the robustness of our results. First, we tested the association of GLP-1 RAs use vs. non-use on outcomes in a 1:5 matched cohort, matched by age, sex, and eGFR category. The analysis was performed by comparing cumulative incidence survival curves using the log-rank test. Second, we matched users and non-users with a 1:5 matching ratio using a propensity score (PS) approach.^19^ We used nearest-neighbour matching without replacement, using a calliper width equal to 0.01 on the logit of the PS. All covariates included in the multivariable Cox regression were used in the PS model. The matching was considered acceptable if the standardized mean difference between treatment groups was <0.1 (Supplementary material online, *Table S1*). The association between GLP-1 RAs use and study outcomes in the PS matched cohort was evaluated both graphically and with an univariable Cox proportional hazard model. The matched analyses do not assume proportional hazards or linearity for the covariates that are being matched on. However, the matched analyses may have low power to detect a difference between GLP-1 RAs use vs. non-use if the hazard ratio (HR) between these groups is highly non-proportional. We assessed the proportional hazards assumption in the matched analyses by Schoenfeld residuals and did not detect any major deviation from proportionality in either of these. Third, the main analysis was performed comparing GLP-1 RAs users and sulfonylurea users, overall and after excluding users of glyburide. Finally, in order to evaluate robustness of our design to bias in misclassification of the exposure, we repeated our main analysis after excluding patients with events occurring within the first 30 days of follow-up. Statistical analysis and data derivation were performed using SAS (SAS Institute Inc., Cary, NC, USA) and R (version 3.4.1).

## Results

### Baseline characteristics

Out of 17 868 patients with diabetes surviving their first MI, 365 (2%) patients were identified as users of GLP-1 RAs. Of these, 316 (86.6%) patients received liraglutide and the remaining 49 received exenatide, lixisenatide, or dulaglutide (7.7%, 4.9%, and 0.8%, respectively). Compared with non-use, patients using GLP-1 RAs were on average younger (median age 65 vs. 71 years), more frequently ex-smokers and more frequently obese (*Table 1*). Users of GLP-1 RAs more often had hypertension or CKD and had undergone PCI. The proportion of patients with Killip >1 was slightly lower among GLP-1 users (8.8% vs. 13.1%) as well as the proportions of users of ACEi/ARBs at discharge.

**Table 1:** Baseline characteristics of diabetes patients at discharge of myocardial infarction

	Overall	No GLP-1 RA users	GLP-1 RA users
Number of individuals	17 868	17 503	365
Age (years)	71 [63, 79]	71 [63, 79]	65 [60, 71]
Age category (years)			
<70	8152 (45.6)	7903 (45.2)	249 (68.2)
≥70	9716 (54.4)	9600 (54.8)	116 (31.8)
Women	6417 (35.9)	6296 (36.0)	121 (33.2)
Smoking			
Never smoker	6804 (38.1)	6689 (38.2)	115 (31.5)
Ex-smoker	6370 (35.7)	6195 (35.4)	175 (47.9)
Current smoker	3407 (19.1)	3347 (19.1)	60 (16.4)
Missing	1287 (7.2)	1272 (7.3)	15 (4.1)
BMI (kg/m^2^)	28.3 [25.3, 31.8]	28.1 [25.3, 31.6]	32.3 [29.4, 35.5]
BMI category (kg/m^2^)			
<30	10 636 (59.5)	10 531 (60.2)	105 (28.8)
≥30	6109 (34.2)	5863 (33.5)	246 (67.4)
Missing	1123 (6.3)	1109 (6.3)	14 (3.8)
eGFR (mL/min/1.73 m^2^)	75 [53, 91]	75 [53, 91]	80 [58, 96]
eGFR category (mL/min/1.73 m^2^)			
<60	5544 (31.0)	5445 (31.1)	99 (27.1)
≥60	11 722 (65.6)	11 468 (65.5)	254 (69.6)
Missing	602 (3.4)	590 (3.4)	12 (3.3)
Comorbidities			
STEMI	5500 (30.8)	5390 (30.8)	110 (30.1)
Heart failure	1613 (9.0)	1583 (9.0)	30 (8.2)
Cancer	727 (4.1)	715 (4.1)	12 (3.3)
Hypertension	13 570 (75.9)	13 261 (75.8)	309 (84.7)
PCI	11 772 (65.9)	11 503 (65.7)	269 (73.7)
CABG	2126 (11.9)	2072 (11.8)	54 (14.8)
Stroke	2350 (13.2)	2316 (13.2)	34 (9.3)
PVD	1148 (6.4)	1131 (6.5)	17 (4.7)
Atrial fibrillation	2500 (14.0)	2458 (14.0)	42 (11.5)
Killip > 1	2321 (13.0)	2289 (13.1)	32 (8.8)
Medications			
Aspirin	16 216 (90.8)	15 879 (90.7)	337 (92.3)
Statins	15 967 (89.4)	15 625 (89.3)	342 (93.7)
ACEi/ARBs	10 331 (57.8)	10 172 (58.1)	159 (43.6)
Beta-blockers	15 769 (88.3)	15 445 (88.2)	324 (88.8)
P2Y12 inhibitors	13 239 (74.1)	12 984 (74.2)	255 (69.9)
Metformin	9551 (53.5)	9387 (53.6)	164 (44.9)
Sulfonylurea	2436 (13.6)	2400 (13.7)	36 (9.9)
DPP4i	1056 (5.9)	1041 (5.9)	15 (4.1)
Insulin	8350 (46.7)	8173 (46.7)	177 (48.5)

ACEi, angiotensin-converting-enzyme inhibitor; ARBs, angiotensin receptor blockers; BMI, Body mass index; CABG, coronary artery bypass grafting; DPP4i, dipeptidyl peptidase 4 inhibitors; eGFR, estimated glomerular filtration rate; GLP-1 RA, GLP-1 receptor agonist; PCI, percutaneous coronary intervention; PVD, peripheral vascular disease; STEMI, ST-segment elevation myocardial infarction.

### Risk of major adverse cardiovascular events

**Figure 1:**
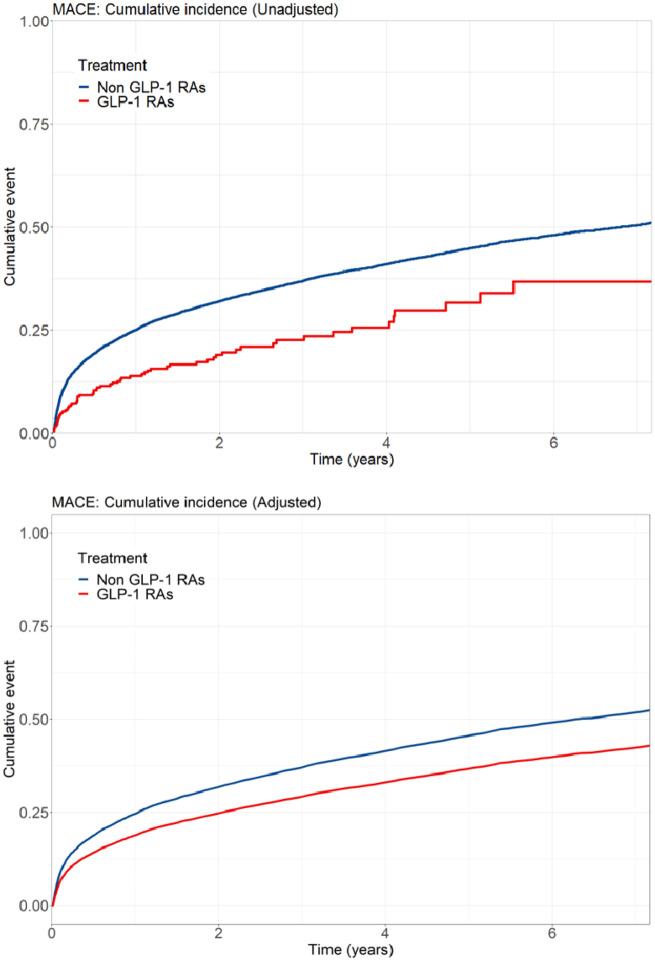
Unadjusted and adjusted cumulative incidence of major adverse cardiovascular event among users and non-users of GLP-1 receptor agonist. GLP-1 RAs, GLP-1 receptor agonists; MACE, major adverse cardiovascular event. Cumulative incidence curve is adjusted for: age, sex, smoking, body mass index, estimated glomerular filtration rate category, comorbidities (heart failure, cancer, hypertension, percutaneous coronary intervention, coronary artery bypass grafting, stroke, peripheral vascular disease, atrial fibrillation, Killip, ST-segment elevation myocardial infarction), and cardiovascular medications (aspirin, statins, angiotensin-converting enzyme inhibitors, angiotensin II receptor blockers, beta-blockers, P2Y12 inhibitors).

**Figure 2:**
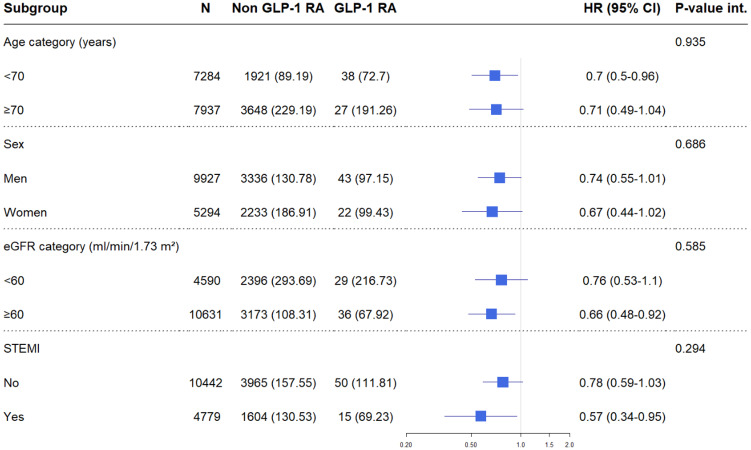
Subgroup analyses: risk of major adverse cardiovascular events among users vs. non-users of GLP-1 receptor agonist by age, sex, ST-segment elevation myocardial infarction (STEMI), and estimated glomerular filtration rate category. CI, confidence interval; eGFR, estimated glomerular filtration rate; HR, hazard ratio; *P*-value int., *P*-value of interaction; STEMI, ST-segment elevation myocardial infarction. Model adjusted for (when relevant): age, sex, smoking, body mass index, estimated glomerular filtration rate category, comorbidities (heart failure, cancer, hypertension, percutaneous coronary intervention, coronary artery bypass grafting, stroke, peripheral vascular disease, atrial fibrillation, Killip, ST-segment elevation myocardial infarction), and cardiovascular medications (aspirin, statins, angiotensin-converting enzyme inhibitors, angiotensin II receptor blockers, beta-blockers, P2Y12 inhibitors).

Among patients fulfilling inclusion and exclusion criteria, 15 221 were considered complete cases with no missing values in any of the covariates employed and included in subsequent analyses (Supplementary material online, *Figure S1* and *Table S1*). During a median follow-up time of 2.98 years, 5634 patients experienced MACE. The cumulative incidence for MACE is shown in *Figure 1*, with lower observed event incidence among GLP-1 RAs users. The event rate among GLP-1 RAs users was lower compared with non-use (97.9 vs. 148.7 per 1000 person-years, respectively). The unadjusted HR associated with GLP-1 RAs use compared with non-use for the composite outcome of MACE was 0.57 [95% confidence interval (CI): 0.45–0.73]. After multivariable adjustment, this association attenuated but remained significantly lower, showing a 28% risk reduction among GLP-1 RAs users (HR: 0.72; 95% CI: 0.56–0.92) (*Table 2*). The direction of the associations for each single MACE component favoured GLP-1 RAs, in particular with stroke and myocardial re-infarction, but it was not statistically significant for CV death (Supplementary material online, *Figure S2*). There was no suggestion of heterogeneity with similar benefit of GLP-1 RAs use in patients with diverse age, sex, eGFR category, or STEMI/NSTEMI (*Figure 2*).

**Table 2:** Risk of cardiovascular events associated with use of GLP-1 RA use vs. non-use

	N events (IR per 1000 PY)	Non GLP-1 RA (IR per 1000 PY)	GLP-1 RA (IR per 1000 PY)	Adjusted HR (95% CI)
MACE (composite)	5634 (147.80)	5569 (148.69)	65 (97.91)	0.72 (0.56–0.92)
Single components of MACE				
Stroke	860 (17.45)	855 (17.63)	5 (6.39)	0.42 (0.18–1.02)
Heart failure	3577 (82.99)	3535 (83.37)	42 (60.25)	0.81 (0.60–1.10)
Myocardial re-infarction	2437 (54.53)	2409 (54.8)	28 (38.34)	0.71 (0.49–1.04)
CV death	1354 (26.56)	1344 (26.78)	10 (12.7)	0.73 (0.39–1.36)

Model adjusted for: age, sex, smoking, body mass index, eGFR category, comorbidities (heart failure, cancer, hypertension, percutaneous coronary intervention, coronary artery bypass grafting, stroke, peripheral vascular disease, atrial fibrillation, Killip, ST-segment elevation myocardial infarction), and cardiovascular medications (aspirin, statins, angiotensin-converting enzyme inhibitors, angiotensin II receptor blockers, beta-blockers, P2Y12 inhibitors).

CI, confidence interval; CV, cardiovascular; GLP-1 RA, GLP-1 receptor agonist; HR, hazard ratio; IR, Incidence rate; MACE, major adverse cardiovascular event; PY, person-years.

As a sensitivity analysis, GLP-1 RAs users were matched with five non-users by age, sex, and eGFR and confirmed a lower cumulative incidence of MACE compared with standard of care (*P*-value of log-rank test = 0.007) (Supplementary material online, *Figure S3*). The 1:5 PS-matching analysis showed similar results than our main analysis, if any stronger in magnitude [HR for MACE 0.61 (0.46–0.80), Supplementary material online, *Table S2*]. Comparisons against users of sulfonylurea showed results in general consistent with our main analysis (Supplementary material online, *Table S3* and *Table* *S4* and  *Figure S4*) for the outcomes of MACE, stroke, HF, and myocardial re-infarction. We note, however, a lack of protection against CV death in GLP1-RAs (HR 1.05; 95% CI: 0.54–2.04) accompanied by broad CIs that rendered the association non-significant*.* Similar results were also shown when we excluded glyburide users from the sulfonylurea group (Supplementary material online, *Table S5*). Finally, excluding patients with events occurring in the first 30 days of follow-up showed results consistent in direction and magnitude to your main analysis (Supplementary material online, *Table S6*). However, broader CIs yielded no statistically significant associations when evaluating the association with single components of MACE.

## Discussion

In this nationwide cohort study of healthcare-managed patients with diabetes surviving an MI, we observe that GLP-1 RAs use in incident MI patients may offer additional cardioprotective benefits over standard of diabetes care. We, thus, expand evidence from clinical trials to routine clinical practice and to patients with recent MI.

Most of the patients in our study used liraglutide as their GLP-1 agent, which represents the market penetration of this drug in Sweden. In this regard, our results are, thus, in line with the analyses from the LEADER trial which showed a CV benefit of liraglutide use compared with placebo, also in patients with history of CVD at inclusion (HR: 0.83; 95% CI: 0.74–0.93).^8^ It also accords with the observational analysis of administrative databases by Svanström *et al*.^20^ showing a lower MACE risk in new users of liraglutide with a history of CVD (HR: 0.81; 95% CI: 0.71–0.92). Both these studies evaluated prevalent CVD patients at baseline, lacking information on the severity of the CV event per se or on the temporality of the association. We, thus, expand current evidence to a homogeneous and well-characterized cohort of survivors of an MI also taking into account relevant information on event severity and underlying kidney function. Our results, however, disagree with the observational analysis of Patorno *et al*.,^21^ who reported no significant difference in the risk of a composite CV outcome (MI, unstable angina, stroke, or coronary revascularization) between users of GLP-1 RAs and DPP-4 inhibitors. We note, however, that 70% of the patients in that study were using exenatide and follow-up was limited to 1 year. In our study, we find interesting the early separation of the survival curves, something that contrasts with the late effect observed in pivotal trials. Although we do not have a clear explanation for this, we speculate that it could be attributed to both the inclusion of prevalent GLP-1 RA users and the possibility of stronger effectiveness during the acute phases of myocardial injury. However, interventional studies to confirm or refute these speculations are so far lacking.

While cardioprotection has also been observed in clinical trials of semaglutide, albiglutide, and dulaglutide,^9^^,^^12^^,^^13^ no CV risk differences were reported in the pivotal trial of exenatide and lixisenatide,^10^^,^^11^ and the associations were less consistent in semaglutide participants from PIONEER-6 trial.^14^ Differences in the temporality of the MI event (i.e. inclusion of patients with a long-term history of MI,^8–10^ or within 90 days from their MI event^13^^,^^14^) or problems with compliance^10^ may explain the differences observed. Nonetheless, meta-analyses of most of these trials suggest collectively that the CV risk reduction is rather consistent, with a meta-estimated 10–13% CV risk reduction overall.^22–24^ Furthermore, we observed no differences in subsequent HF hospitalization among GLP-1 RA users, something that agrees with recent meta-analysis on liraglutide pivotal trials^25^ and in a *post* *hoc* analysis of the EXSCEL-trial.^26^ Given the predominance of liraglutide use in our setting, we were unable to compare single GLP-1 agents.

Subgroup analyses show similar results across age strata, sex, and severity of the MI event. Furthermore, across the kidney function spectrum enrolled, there was no consistent effect modification by the presence or absence of chronic kidney disease (eGFR < 60 mL/min/1.73 m^2^). This again is in agreement with *post* *hoc* analyses from LEADER showing consistent MACE reduction regardless presence of chronic kidney disease^27^ and may represent an interesting alternative for patients with diabetic kidney disease, especially in view of plausible effects of GLP-1 use in retarding chronic kidney disease progression.^28^^,^^29^ Diabetic kidney disease develops frequently among patients with diabetes, but the majority of them die from CV causes and infections before needing kidney replacement therapy.^30^ We were unable to study kidney replacement therapy as an outcome, because although we identified about 200 events in our cohort, only two events occurred among GLP-1 RA users.

Although the mechanism through which some of the GLP-1 RAs reduce MACE is not clear, the cardio-protective effects seem to go beyond glucose control,^31^ possibly involving weight loss, as well as improved insulin resistance and lipid levels.^32–36^ Pre-clinical studies have shown a reduction of atherosclerosis formation by GLP-1 RAs through inhibition of plaque progression and promoting plague stabilization^37^; GLP-1 RAs may reduce systemic and vascular inflammation by lowering the expression of inflammatory proteins involved in the development and progression of atherosclerosis^38^; and finally, studies have suggested a dose-dependent effect between GLP-1 RAs and changes in BP by increasing electrolytes excretion^39^^,^^40^ and reducing the circulating levels of RAS system components.^41^^,^^42^ However, not all of these mechanisms have been confirmed in clinical trials and warrant further characterization.

Our study has some strengths and limitations. Strengths are the rich quality of information collected from a nationwide register in a country with universal healthcare and medication costs subsidize by the government. This may reduce biases due to socio-economic differences or access to healthcare. Limitations include our inability to establish causality in the observed associations and to adequately separate prior use vs. post-MI initiation of GLP-1 RAs. Drug exposure status was identified from filled prescriptions; non-use of dispensed drugs would lead to exposure misclassification. The primary intention-to-treat exposure definition allowed for complete follow-up and estimation of the overall impact of starting treatment with GLP-1 RA, but might also have resulted in exposure misclassification. Given the drug indication, we can safely assume that all GLP1-RAs had type 2 diabetes. However, we could not differentiate type 1 from type 2 diabetes reliably, and some non-users may have type 1. This being said, previous validation studies in SWEDEHEART evidence that <5% of participants had type 1 diabetes,^43^ which argues against relevant misclassification bias. We lack information on important covariates such as HbA1c and diabetes duration. As in any observational study, and despite the application of several key measures to reduce the potential for bias—including use of an active comparator, and PS matching—confounding from unmeasured risk factors cannot be ruled out. We have a relatively low number of GLP-1 RAs users in our study. However, these are representative of the Swedish population during 2010–17. In any case, extrapolation to other periods and countries should be done with caution. Nonetheless, considering the very limited evidence to date on GLP-1 RA use in a near MI-event, our data are of value in reporting at least no adverse events in this high-risk patient group. From a patient selection point of view, it may be argued that if GLP-1 RA is effective in primary CV prevention,^8^^,^^9^ our incident MI cases represent a selective patient population in which the GLP-1 RA treatment was less effective. If true, this would have brought our estimates towards the null.

We conclude that compared with the standard of diabetes care, the use of GLP-1 RA by routinely cared survivors of an acute MI was associated with a lower risk of subsequent major CV adverse events.

## Supplementary material

Supplementary material is available at *European Heart Journal – Cardiovascular Pharmacotherapy* online.

**Conflict of interest:** J.J.C. reports receiving funding from AstraZeneca, Viforpharma, Astellas, and Novartis for projects unrelated to this study. T.J. reports research grants from Novartis and Merck and fees for lecturing and consulting from Astra-Zeneca, Aspen, Bayer, Merck, Novartis, and Sanofi. A.N. reports receiving consulting and lectures fees from AstraZeneca, Novo Nordisk, Boehringer Ingelheim, and Merck in Sweden all unrelated to this study. P.L. reports receiving consulting and lectures fees from AstraZeneca, Amgen, Boehringer Ingelheim, and Sanofi in Sweden all unrelated to this study. The rest of the co-authors have no conflicts of interest to report.

## Supplementary Material

pvaa004_Supplementary_Data
