# Supplementary material for: Glucagon-like peptide-1 receptor agonists and the risk of cardiovascular events in diabetes patients surviving an acute myocardial infarction
Source: Eur Heart J Cardiovasc Pharmacother. 2020 Jan 30;7(2):104–11. doi: 10.1093/ehjcvp/pvaa004 (PMC7957901; doi:10.1093/ehjcvp/pvaa004)
Supplement: pvaa004_Supplementary_Data [file pvaa004_supplementary_data.docx]

**Supplemental material**

**Glucagon-like peptide-1 receptor agonists and the risk of cardiovascular events in diabetes patients surviving an acute myocardial infarction.**

Contents

[Table S1. Baseline characteristics before and after propensity score matching 2](#_Toc26125523)

[Table S2. Risk of cardiovascular events associated with GLP-1 RA use in the 1:5 propensity score matched cohort 3](#_Toc26125524)

[Table S3. Baseline characteristics of GLP-1 RA and sulfonylurea users 4](#_Toc26125525)

[Table S4. Risk of cardiovascular outcomes in GLP-1 RA versus sulfonylurea users 5](#_Toc26125526)

[Table S5. Risk of cardiovascular outcomes in GLP-1 RA versus sulfonylurea users excluding glyburide users 6](#_Toc26125527)

[Table S6. Risk of cardiovascular outcomes in GLP-1 RA use vs non-use among patients that survived event-free in the first 30 days of follow-up. 7](#_Toc26125528)

[Figure S1. Flow-chart 8](#_Toc26125529)

[Figure S2. Survival probability of cardiovascular mortality by treatment group 9](#_Toc26125530)

[Figure S3. Cumulative incidence of major adverse cardiovascular events (MACE) and survival probability of cardiovascular death in the 1:5 cohort matched by age, sex and eGFR category 10](#_Toc26125531)

[Figure S4. Risk of MACE in GLP-1 RA versus sulfonylurea users by age, sex, ST elevation myocardial infarction (STEMI) and estimated glomerular filtration rate (eGFR) category 11](#_Toc26125532)

# Table S1. Baseline characteristics before and after propensity score matching

|  | Before PS matching | | | After PS matching | | |
| --- | --- | --- | --- | --- | --- | --- |
|  | Non GLP-1 RA | GLP-1 RA | SMD | Non GLP-1 RA | GLP-1 RA | SMD |
| Number of individuals | 14894 | 327 |  | 1420 | 284 |  |
| Age (years) | 70  [62, 78] | 65  [60, 71] | 0.443 | 67  [58, 74] | 67  [60, 72] | 0.038 |
| Female | 5188 (34.8) | 106 (32.4) | 0.051 | 468 (33.0) | 95 (33.5) | 0.010 |
| Smoking status |  |  | 0.253 |  |  | 0.032 |
| Never smoker | 6047 (40.6) | 107 (32.7) |  | 490 (34.5) | 101 (35.6) |  |
| ex-smoker | 5752 (38.6) | 167 (51.1) |  | 664 (46.8) | 133 (46.8) |  |
| current smoker | 3095 (20.8) | 53 (16.2) |  | 266 (18.7) | 50 (17.6) |  |
| BMI category (kg/m2) |  |  | 0.722 |  |  | 0.064 |
| <30 | 9514 (63.9) | 98 (30.0) |  | 447 (31.5) | 98 (34.5) |  |
| ≥30 | 5380 (36.1) | 229 (70.0) |  | 973 (68.5) | 186 (65.5) |  |
| eGFR (ml/min/1.73 m^2^) | 77  [55, 92] | 80  [58, 96] | 0.145 | 80  [58, 94] | 78  [57, 95] | 0.006 |
| *Comorbidities* | | | | | | |
| STEMI | 5390 (30.8) | 110 (30.1) | 0.015 | 516 (30.6) | 103 (30.6) | 0.001 |
| Heart failure | 1583 (9.0) | 30 (8.2) | 0.029 | 132 (7.8) | 28 (8.3) | 0.017 |
| Cancer | 715 (4.1) | 12 (3.3) | 0.042 | 53 (3.1) | 12 (3.6) | 0.023 |
| Hypertension | 13261 (75.8) | 309 (84.7) | 0.225 | 1427 (84.7) | 281 (83.4) | 0.036 |
| PCI | 11503 (65.7) | 269 (73.7) | 0.174 | 1240 (73.6) | 246 (73.0) | 0.013 |
| CABG | 2072 (11.8) | 54 (14.8) | 0.087 | 247 (14.7) | 48 (14.2) | 0.012 |
| Stroke | 2316 (13.2) | 34 (9.3) | 0.124 | 175 (10.4) | 33 (9.8) | 0.020 |
| PVD | 1131 (6.5) | 17 (4.7) | 0.079 | 82 (4.9) | 16 (4.7) | 0.006 |
| Atrial fibrillation | 2458 (14.0) | 42 (11.5) | 0.076 | 212 (12.6) | 40 (11.9) | 0.022 |
| Killip > 1 | 2289 (13.1) | 32 (8.8) | 0.139 | 139 (8.2) | 32 (9.5) | 0.044 |
| *Medications* | | | | | | |
| Aspirin | 15879 (90.7) | 337 (92.3) | 0.058 | 1562 (92.7) | 313 (92.9) | 0.007 |
| Statins | 15625 (89.3) | 342 (93.7) | 0.159 | 1572 (93.3) | 315 (93.5) | 0.007 |
| ACEi/ARBs | 10172 (58.1) | 159 (43.6) | 0.294 | 772 (45.8) | 156 (46.3) | 0.010 |
| Beta-blockers | 15445 (88.2) | 324 (88.8) | 0.016 | 1492 (88.5) | 299 (88.7) | 0.006 |
| P2Y12 inhibitors | 12984 (74.2) | 255 (69.9) | 0.096 | 1200 (71.2) | 243 (72.1) | 0.020 |

Abbreviations: GLP-1 RA, GLP-1 receptor agonist; BMI, Body mass index; eGFR, estimated glomerular filtration rate; STEMI, ST-segment elevation myocardial infarction; PCI, percutaneous coronary intervention; CABG, coronary artery bypass grafting; PVD, peripheral vascular disease; ACEi, angiotensin-converting-enzyme inhibitor; ARBs, angiotensin receptor blockers; DPP4i, dipeptidyl peptidase 4 inhibitors

# Table S2. Risk of cardiovascular events associated with GLP-1 RA use in the 1:5 propensity score matched cohort

|  | **N events**  **(IR per 1000 PY)** | **Non GLP-1 RA**  **(IR per 1000 PY)** | **GLP-1 RA**  **(IR per 1000 PY)** | **Adjusted HR**  **(95% CI)** |
| --- | --- | --- | --- | --- |
| **MACE (composite)** | 541 (128.11) | 487 (133.51) | 54 (93.84) | 0.61 ( 0.46 - 0.80 ) |
| **Single components of MACE** | | | | |
| **Stroke** | 80 (14.77) | 76 (16.05) | 4 (5.89) | 0.35 ( 0.13 - 0.95 ) |
| **Heart failure** | 357 (75.24) | 322 (77.78) | 35 (57.9) | 0.63 ( 0.45 - 0.90 ) |
| **Myocardial re-infarction** | 243 (49.63) | 219 (51.31) | 24 (38.22) | 0.64 ( 0.42 - 0.98 ) |
| **CV death** | 97 (17.4) | 88 (17.98) | 9 (13.22) | 0.65 ( 0.33 - 1.28 ) |

Abbreviations: GLP-1 RA, GLP-1 receptor agonist; IR, Incidence rate; PY, person-year; HR, hazard ratio; CI, confidence interval; MACE, major adverse cardiovascular event; CV, cardiovascular

# Table S3. Baseline characteristics of GLP-1 RA and sulfonylurea users

|  | **Overall** | **Sulfonylurea** | **GLP-1 RA users** | |
| --- | --- | --- | --- | --- |
| Number of individuals | 2729 | 2400 | 329 | |
| Age (years) | 73 [65, 81] | 74 [66, 82] | 66 [59, 72] | |
| Age category (years) |  |  |  | |
| < 70 | 1071 (39.2) | 852 (35.5) | 219 (66.6) | |
| ≥ 70 | 1658 (60.8) | 1548 (64.5) | 110 (33.4) | |
| Women | 1028 (37.7) | 919 (38.3) | 109 (33.1) | |
| Smoking |  |  |  | |
| Never smoker | 1097 (40.2) | 995 (41.5) | 102 (31.0) | |
| ex-smoker | 970 (35.6) | 813 (33.9) | 157 (47.7) | |
| current smoker | 457 (16.8) | 402 (16.8) | 55 (16.7) | |
| missing | 205 (7.5) | 190 (7.9) | 15 (4.6) | |
| BMI (kg/m^2^) | 28.1 [25.2, 31.6] | 27.6 [24.8, 30.8] | 32.1 [29.5, 35.4] | |
| BMI category (kg/m^2^) |  |  |  | |
| <30 | 1647 (60.4) | 1553 (64.7) | 94 (28.6) | |
| ≥30 | 890 (32.6) | 668 (27.8) | 222 (67.5) | |
| eGFR (ml/min/1.73 m^2^) | 74 [53, 90] | 73 [53, 89] | 79 [56, 96] | |
| eGFR category (ml/min/1.73 m^2^) |  |  |  | |
| <60 | 877 (32.1) | 783 (32.6) | 94 (28.6) | |
| ≥60 | 1756 (64.3) | 1532 (63.8) | 224 (68.1) | |
| missing | 96 (3.5) | 85 (3.5) | 11 (3.3) | |
| Comorbidities | | | |  |
| STEMI | 791 (29.0) | 692 (28.8) | 99 (30.1) | |
| Heart failure | 239 (8.8) | 212 (8.8) | 27 (8.2) | |
| Cancer | 87 (3.2) | 75 (3.1) | 12 (3.6) | |
| Hypertension | 2154 (78.9) | 1874 (78.1) | 280 (85.1) | |
| PCI | 1734 (63.5) | 1493 (62.2) | 241 (73.3) | |
| CABG | 321 (11.8) | 271 (11.3) | 50 (15.2) | |
| Stroke | 383 (14.0) | 354 (14.8) | 29 (8.8) | |
| PVD | 163 (6.0) | 147 (6.1) | 16 (4.9) | |
| Atrial fibrillation | 422 (15.5) | 385 (16.0) | 37 (11.2) | |
| Killip > 1 | 386 (14.1) | 358 (14.9) | 28 (8.5) | |
| Medications | | | |  |
| Aspirin | 2492 (91.3) | 2191 (91.3) | 301 (91.5) | |
| Statins | 2364 (86.6) | 2054 (85.6) | 310 (94.2) | |
| ACEi/ARBs | 1539 (56.4) | 1403 (58.5) | 136 (41.3) | |
| Beta-blockers | 2405 (88.1) | 2115 (88.1) | 290 (88.1) | |
| P2Y12 inhibitors | 2004 (73.4) | 1772 (73.8) | 232 (70.5) | |
| Metformin | 1301 (47.7) | 1157 (48.2) | 144 (43.8) | |
| DPP4i | 178 (6.5) | 164 (6.8) | 14 (4.3) | |
| Insulin | 633 (23.2) | 469 (19.5) | 164 (49.8) | |

Abbreviations: GLP-1 RA, GLP-1 receptor agonist; BMI, Body mass index; eGFR, estimated glomerular filtration rate; STEMI, ST-segment elevation myocardial infarction; PCI, percutaneous coronary intervention; CABG, coronary artery bypass grafting; PVD, peripheral vascular disease; ACEi, angiotensin-converting-enzyme inhibitor; ARBs, angiotensin receptor blockers; DPP4i, dipeptidyl peptidase 4 inhibitors

# Table S4. Risk of cardiovascular outcomes in GLP-1 RA versus sulfonylurea users

|  | **N events**  **(IR per 1000 PY)** | **Sulfonylurea**  **(IR per 1000 PY)** | **GLP-1 RA**  **(IR per 1000 PY)** | **Adjusted HR**  **(95% CI) ^1^** |
| --- | --- | --- | --- | --- |
| **MACE (composite)** | 911 (154.99) | 849 (160.12) | 62 (107.68) | 0.77 ( 0.58 – 1.00 ) |
| **Single components of MACE** | | | | |
| **Stroke** | 145 (18.67) | 140 (19.78) | 5 (7.25) | 0.49 ( 0.20 - 1.22 ) |
| **Heart failure** | 567 (83.93) | 526 (85.53) | 41 (67.7) | 0.93 ( 0.66 - 1.29 ) |
| **Myocardial re-infarction** | 395 (56.8) | 369 (58.45) | 26 (40.57) | 0.69 ( 0.46 - 1.05 ) |
| **CV death** | 239 (29.75) | 229 (31.2) | 10 (14.39) | 1.05 ( 0.54 - 2.04 ) |

Abbreviations: GLP-1 RA, GLP-1 receptor agonist; IR, Incidence rate; PY, person-year; HR, hazard ratio; CI, confidence interval; MACE, major adverse cardiovascular event; CV, cardiovascular

Model adjusted for: age, sex, smoking, body mass index, eGFR category, comorbidities (heart failure, cancer, hypertension, percutaneous coronary intervention, coronary artery bypass grafting, stroke, peripheral vascular disease, atrial fibrillation, killip, ST-segment elevation myocardial infarction) and cardiovascular medications (aspirin, statins, angiotensin-converting enzyme inhibitors, angiotensin II receptor blockers, beta blockers, P2Y12 inhibitors)

# Table S5. Risk of cardiovascular outcomes in GLP-1 RA versus sulfonylurea users excluding glyburide users

|  | **N events**  **(IR per 1000 PY)** | **Sulfonylurea**  **(N = 1291)**  **(IR per 1000 PY)** | **GLP-1 RA**  **(N = 293)**  **(IR per 1000 PY)** | **Adjusted HR**  **(95% CI)** |
| --- | --- | --- | --- | --- |
| **MACE (composite)** | 576 (152.16) | 514 (160.13) | 62 (107.68) | 0.73 ( 0.55 - 0.97 ) |
| **Single components of MACE** | | | | |
| **Stroke** | 80 (16.07) | 75 (17.48) | 5 (7.25) | 0.66 ( 0.26 - 1.73 ) |
| **Heart failure** | 368 (85.44) | 327 (88.35) | 41 (67.7) | 0.83 ( 0.59 - 1.19 ) |
| **Myocardial re-infarction** | 248 (55.64) | 222 (58.18) | 26 (40.57) | 0.66 ( 0.43 - 1.02 ) |
| **CV death** | 133 (25.99) | 123 (27.82) | 10 (14.39) | 1.05 ( 0.52 - 2.11 ) |

Abbreviations: GLP-1 RA, GLP-1 receptor agonist; IR, Incidence rate; PY, person year; HR, hazard ratio; CI, confidence interval; MACE, major adverse cardiovascular event; CV, cardiovascular

Model adjusted for: age, sex, smoking, body mass index, eGFR category, comorbidities (heart failure, cancer, hypertension, percutaneous coronary intervention, coronary artery bypass grafting, stroke, peripheral vascular disease, atrial fibrillation, killip, ST-segment elevation myocardial infarction) and cardiovascular medications (aspirin, statins, angiotensin-converting enzyme inhibitors, angiotensin II receptor blockers, beta blockers, P2Y12 inhibitors)

# Table S6. Risk of cardiovascular outcomes in GLP-1 RA use vs non-use among patients that survived event-free in the first 30 days of follow-up.

|  | **N events**  **(IR per 1000 PY)** | **Non GLP-1 RA**  **(N = 16,908)**  **(IR per 1000 PY)** | **GLP-1 RA**  **(N = 355)**  **(IR per 1000 PY)** | **Adjusted HR**  **(95% CI)** |
| --- | --- | --- | --- | --- |
| **MACE (composite)** | 4921 (126.74) | 4868 (127.49) | 53 (82.23) | 0.76 ( 0.58 - 0.99 ) |
| **Single components of MACE** | | | | |
| **Stroke** | 809 (16.88) | 804 (17.04) | 5 (6.66) | 0.46 ( 0.19 - 1.12 ) |
| **Heart failure** | 3266 (76.75) | 3229 (77.1) | 37 (55.25) | 0.88 ( 0.63 - 1.22 ) |
| **Myocardial re-infarction** | 1950 (43.29) | 1927 (43.46) | 23 (32.34) | 0.82 ( 0.54 - 1.24 ) |
| **CV death** | 1149 (23.22) | 1142 (23.44) | 7 (9.27) | 0.62 ( 0.3 - 1.32 ) |

Abbreviations: GLP-1 RA, GLP-1 receptor agonist; IR, Incidence rate; PY, person-year; HR, hazard ratio; CI, confidence interval; MACE, major adverse cardiovascular event; CV, cardiovascular

Model adjusted for: age, sex, smoking, body mass index, eGFR category, comorbidities (heart failure, cancer, hypertension, percutaneous coronary intervention, coronary artery bypass grafting, stroke, peripheral vascular disease, atrial fibrillation, killip, ST-segment elevation myocardial infarction) and cardiovascular medications (aspirin, statins, angiotensin-converting enzyme inhibitors, angiotensin II receptor blockers, beta blockers, P2Y12 inhibitors)

# Figure S1. Flow-chart


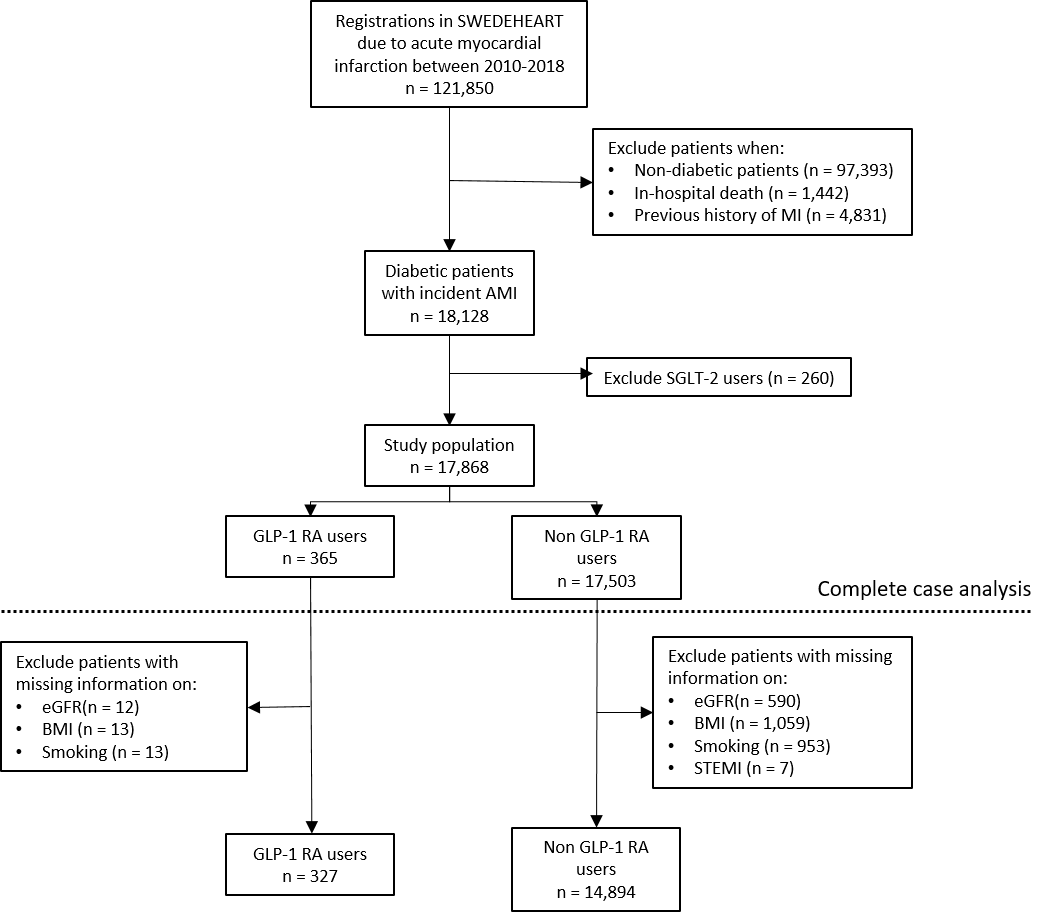


# Figure S2. Survival probability of cardiovascular mortality by treatment group


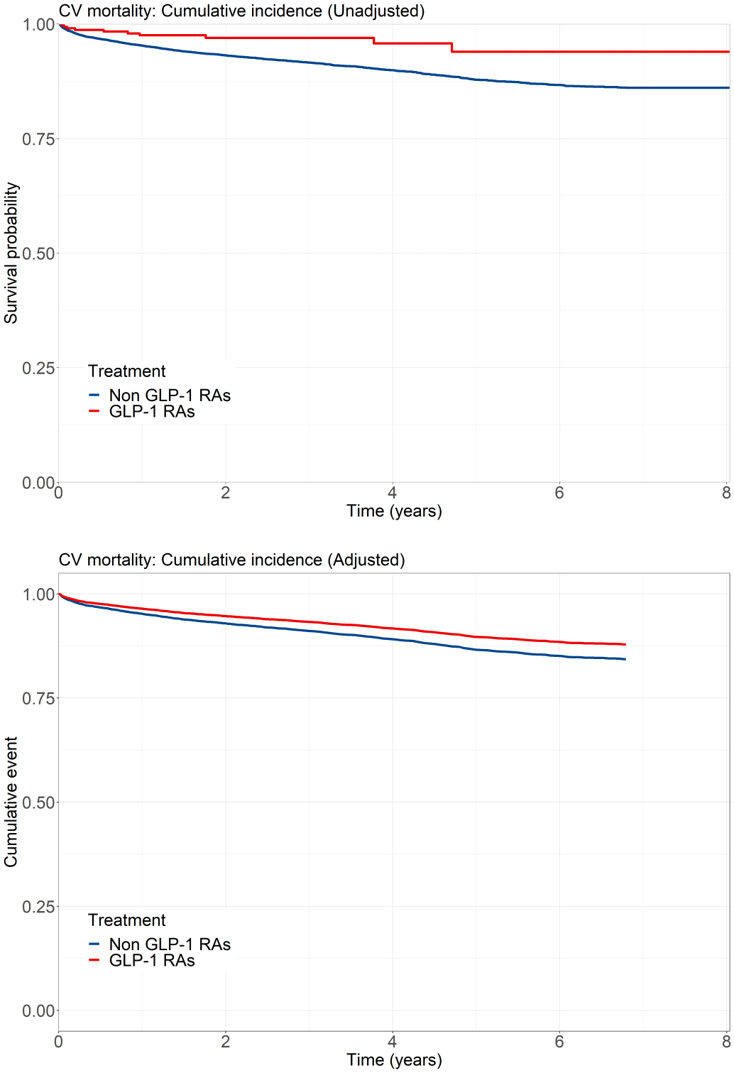


Abbreviations: GLP-1 RAs, GLP-1 receptor agonists; CV, cardiovascular

Adjusted for: age, sex, smoking, body mass index, eGFR category, comorbidities (heart failure, cancer, hypertension, percutaneous coronary intervention, coronary artery bypass grafting, stroke, peripheral vascular disease, atrial fibrillation, killip, ST-segment elevation myocardial infarction) and cardiovascular medications (aspirin, statins, angiotensin-converting enzyme inhibitors, angiotensin II receptor blockers, beta blockers, P2Y12 inhibitors)

# Figure S3. Cumulative incidence of major adverse cardiovascular events (MACE) and survival probability of cardiovascular death in the 1:5 cohort matched by age, sex and eGFR category


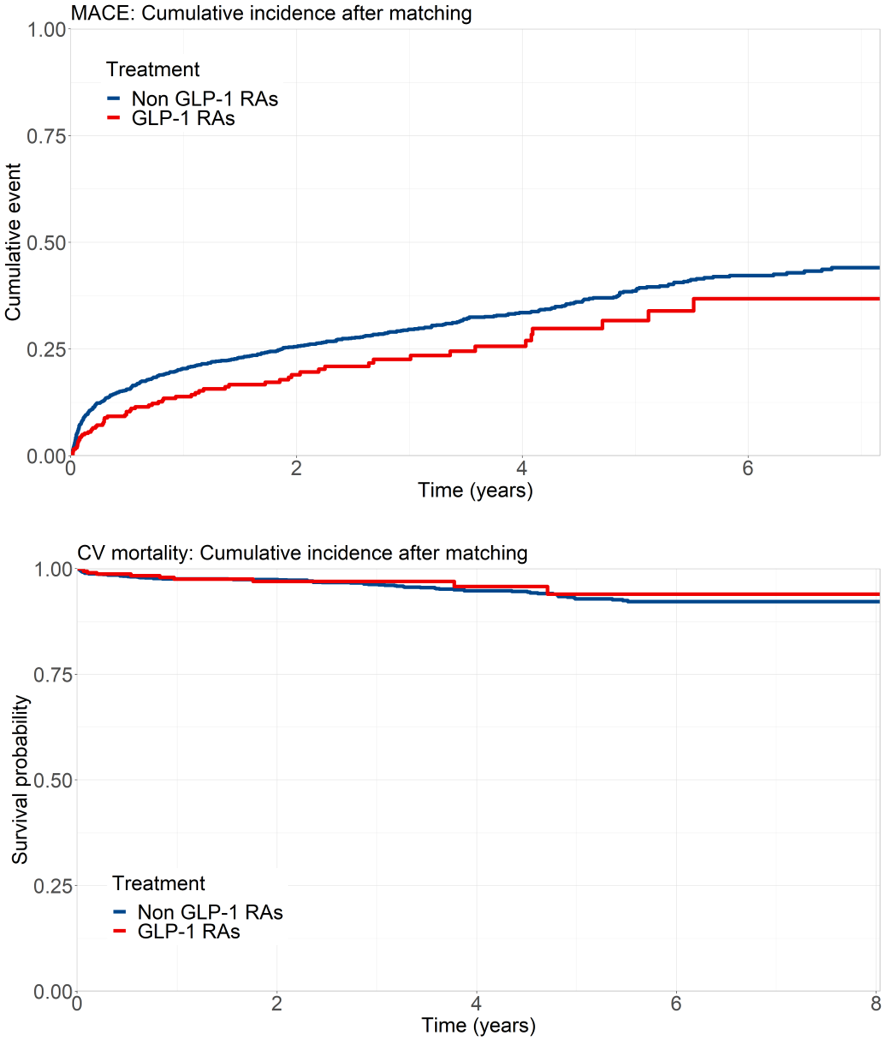


Abbreviations: GLP-1 RAs, GLP-1 receptor agonists; MACE, major adverse cardiovascular event; CV, cardiovascular

# Figure S4. Risk of MACE in GLP-1 RA versus sulfonylurea users by age, sex, ST elevation myocardial infarction (STEMI) and estimated glomerular filtration rate (eGFR) category


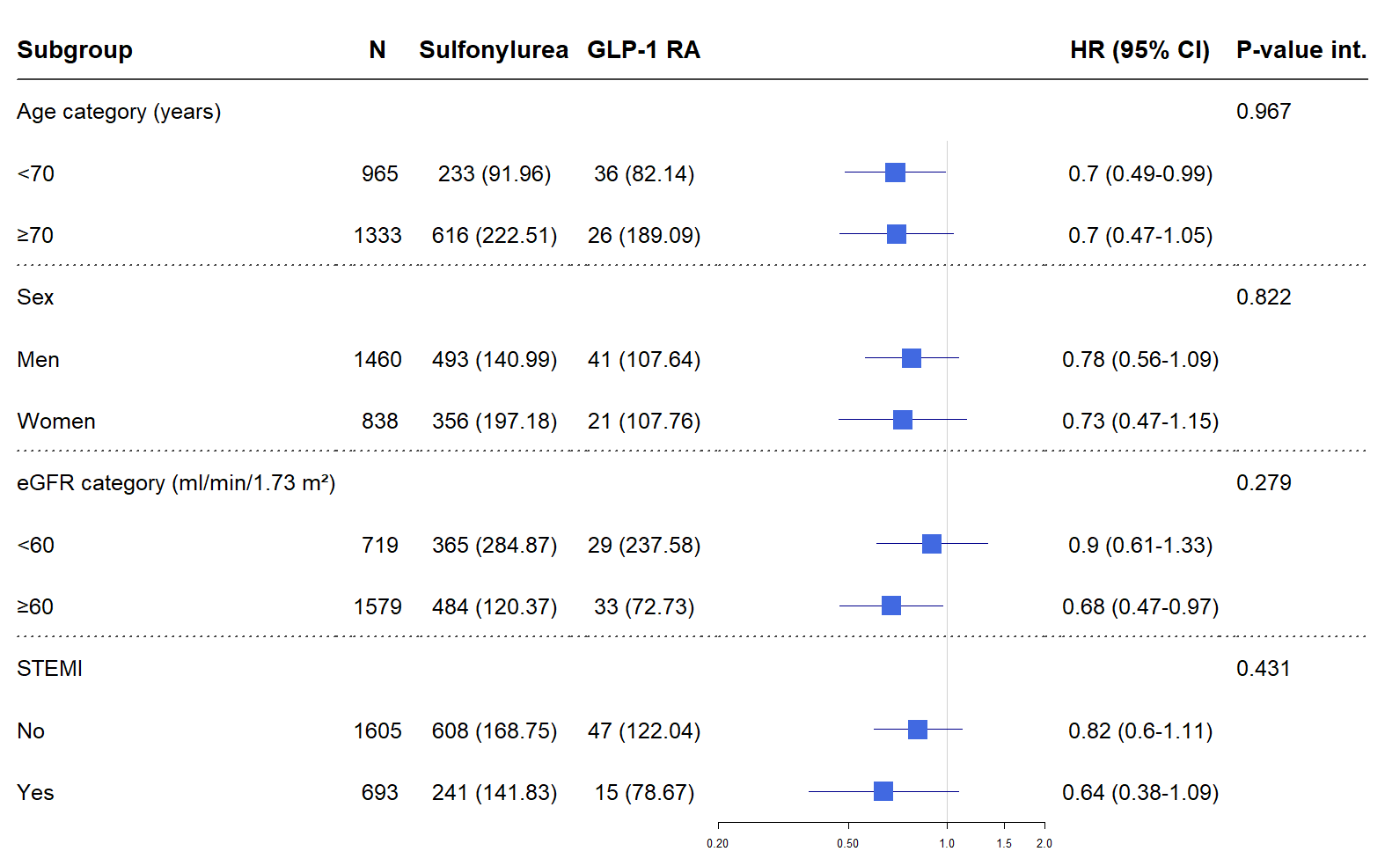


Abbreviations: GLP-1 RA, GLP-1 receptor agonist; HR, hazard ratio; CI, confidence interval; eGFR, estimated glomerular filtration rate; STEMI, ST-segment elevation myocardial infarction; P-value int., p-value interaction

Model adjusted for (when relevant): age, sex, smoking, body mass index, eGFR category, comorbidities (heart failure, cancer, hypertension, percutaneous coronary intervention, coronary artery bypass grafting, stroke, peripheral vascular disease, atrial fibrillation, killip, ST-segment elevation myocardial infarction) and cardiovascular medications (aspirin, statins, angiotensin-converting enzyme inhibitors, angiotensin II receptor blockers, beta blockers, P2Y12 inhibitors)
